# PKMζ is essential for spinal plasticity underlying the maintenance of persistent pain

**DOI:** 10.1186/1744-8069-7-99

**Published:** 2011-12-20

**Authors:** Andre Laferrière, Mark H Pitcher, Anne Haldane, Yue Huang, Virginia Cornea, Naresh Kumar, Todd C Sacktor, Fernando Cervero, Terence J Coderre

**Affiliations:** 1 Department of Anesthesia, McGill University, 3655 Promenade Sir William Osler, Montreal, Quebec, H3G 1Y6, Canada; 2 Alan Edwards Centre for Research on Pain, McGill University, 740 Dr. Penfield Ave., Montreal, Quebec, H3A 1A4, Canada; 3 Departments of Neurology & Neurosurgery, McGill University, 3801 University St., Montreal Quebec H3A 2B4, Canada; 4 Department of Psychology, McGill University, 1205 Dr. Penfield Ave., Montreal, Quebec, H3A 1B1, Canada; 5 Departments of Physiology, Pharmacology, and Neurology, State University of New York Downstate Medical Center, 450 Clarkson Avenue, Brooklyn, New York 11203, USA; 6 Faculty of Dentistry, McGill University, 3640 University St., Montreal, Quebec, H3A 2B2, Canada; 7 McGill University Hospital Centre Research Institute, 2155 Guy St., Montreal, Quebec, H3H 2R9, Canada

**Keywords:** nociception, protein kinase C, central nociceptive sensitization

## Abstract

**Background:**

Chronic pain occurs when normally protective acute pain becomes pathologically persistent. We examined here whether an isoform of protein kinase C (PKC), PKMζ, that underlies long-term memory storage in various brain regions, also sustains nociceptive plasticity in spinal cord dorsal horn (SCDH) mediating persistent pain.

**Results:**

Cutaneous injury or spinal stimulation produced persistent increases of PKMζ, but not other atypical PKCs in SCDH. Inhibiting spinal PKMζ, but not full-length PKCs, reversed plasticity-dependent persistent painful responses to hind paw formalin and secondary mechanical hypersensitivity and SCDH neuron sensitization after hind paw capsaicin, without affecting peripheral sensitization-dependent primary heat hypersensitivity after hind paw capsaicin. Inhibiting spinal PKMζ, but not full-length PKCs, also reversed mechanical hypersensitivity in the rat hind paw induced by spinal stimulation with intrathecal dihydroxyphenylglycine. Spinal PKMζ inhibition also alleviated allodynia 3 weeks after ischemic injury in rats with chronic post-ischemia pain (CPIP), at a point when allodynia depends on spinal changes. In contrast, spinal PKMζ inhibition did not affect allodynia in rats with chronic contriction injury (CCI) of the sciatic nerve, or CPIP rats early after ischemic injury, when allodynia depends on ongoing peripheral inputs.

**Conclusions:**

These results suggest spinal PKMζ is essential for the maintenance of persistent pain by sustaining spinal nociceptive plasticity.

## Background

Chronic pain follows the transition from normally protective acute pain to pathological persistent pain, and depends on neuronal plasticity in SCDH [1,2]. Protein kinases, such as PKC, contribute to nociceptive plasticity in SCDH [3]. Specific isoforms of PKC, including PKCβ-II, PKCε, PKCγ and PKCζ are upregulated in SCDH after persistent pain [4-8], and persistent pain is relieved by inhibition/knock-out of these isoforms [9-11]. Although studies implicate full-length PKCs in the induction of spinal nociceptive plasticity, the mechanisms that sustain nociceptive plasticity underlying persistent pain are unknown.

An independent C-terminal domain of PKCζ, known as PKMζ, exists as an autonomously-active isoform. PKMζ is generated by an internal promoter within the PKCζ gene that encodes only the catalytic domain [12]. New synthesis of PKMζ is necessary and sufficient for maintenance of hippocampal long-term potentiation (LTP) [13,14], and long-term memory storage in various brain regions [15-17]. Given there are similarities in the neuronal mechanisms underlying both hippocampal LTP and spinal nociceptive plasticity [18], including a key role for PKC in hippocampal LTP [19,20], we expect that PKMζ may also contribute to the maintenance of spinal plasticity underlying persistent nociception.

A recent study shows that neuropathic pain in mice is reduced by inhibiting PKMζ in cingulate cortex, but not SCDH [21]. However, since the specific contribution of PKMζ to the maintenance of plasticity in neuropathic pain is confounded by ongoing peripheral inputs, it is important to assess the effects of PKMζ inhibition in an animal model of persistent pain, where allodynia depends on central changes independent of ongoing peripheral inputs.

PKMζ has also been shown to contribute to spinal nociceptive priming [22], although it is unknown whether PKMζ maintains spinal plasticity allowing acute stimuli to generate persistent pain, clinically a critical step in progression from acute to chronic pain [23,24]. Furthermore, while inhibitors of full-length PKCs have been shown to be involved in the induction of nociceptive sensitization in SCDH neurons [25,26], it is unknown whether PKMζ contributes to the maintenance of this sensitization.

Here we examine whether PKMζ is selectively involved in sustaining spinal plasticity essential for persistent pain, and underlies persistent pain, pain hypersensitivity and/or SCDH neuronal sensitization following cutaneous injury or direct spinal stimulation. We also examine whether PKMζ inhibition selectively reverses chronic allodynia that depends on central changes, as opposed to ongoing peripheral inputs.

## Results

### Spinal PKMζ is upregulated after cutaneous injury

**Figure 1:**
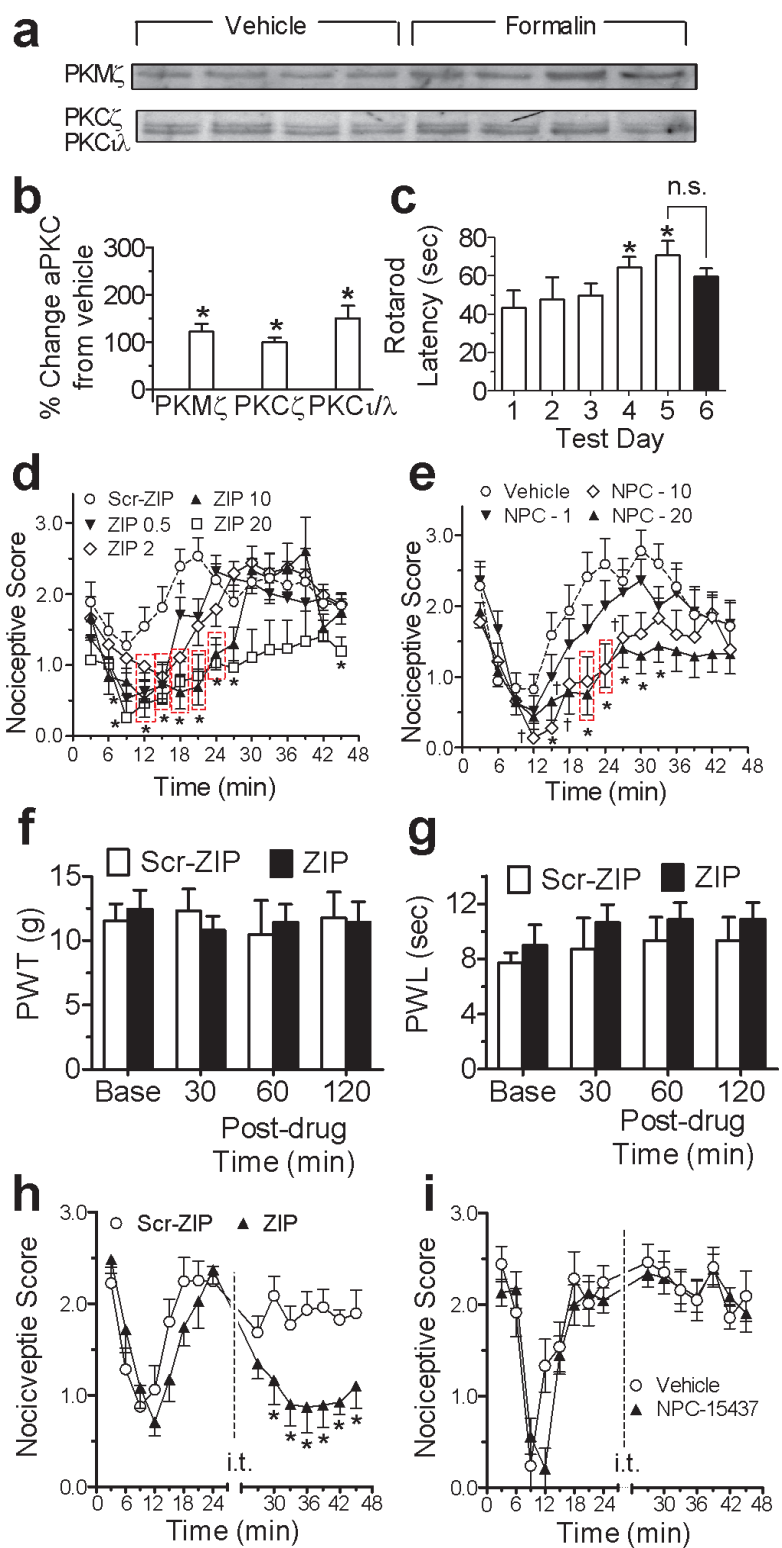
***SCDH atypical PKC expression, and effects of PKMζ/PKC inhibition on formalin nociception***. (**a**) Western blots and (**b**) histograms (% change) of atypical PKC showing i.pl. formalin increased PKMζ, PKCζ and PKCι/λ (n = 5/6 for vehicle/formalin) in SCDH (*P < 0.05). (**c**) Effect of ZIP on rotarod latencies, which significantly increased over training (*p < 0.05 from day 1). Latencies on day 6 (after ZIP) did not differ from day 5 (n.s.: non-significant difference). (**d,e**) Dose-dependent effects of *pretreatment *with ZIP (n = 5-7/dose) vs. scr-ZIP (n = 7) (**d**) or NPC-15437 (n = 5-7/dose) vs. vehicle (n = 8) (**e**) on formalin pain. ZIP dose-dependently reduced late phase (9-45 min) scores (†P < 0.05, *P < 0.01 red dash-boxed or individual symbols from scr-ZIP), but not early phase scores (0-6 min). NPC-15437 also significantly reduced late phase scores (†P < 0.05, *P < 0.01 for red dash-boxed or individual symbols), but not early phase scores. (**f,g**) Effect of ZIP (n = 6) or scr-ZIP (n = 4) pretreatment on von Frey paw-withdrawal thresholds (PWT, **f**) or plantar test paw-withdrawal latencies (PWL, **g**) in naïve rats. Neither ZIP nor scr-ZIP significantly affected either PWTs or PWLs. (**h,i**) Painful responses induced by formalin before and after ZIP (n = 5) or scr-ZIP (n = 5) (**h**), and NPC-15437 (n = 5) or vehicle (n = 7) (**i**), administered 25 min *post-formalin*. ZIP (*P <.05), but not NPC-15437, significantly attenuated persistent formalin pain. For these and subsequent graphs ZIP, scr-ZIP, NPC-15437 and vehicle were given i.t., and data is expressed as means ± s.e.m. unless otherwise stated.

**Figure 2:**
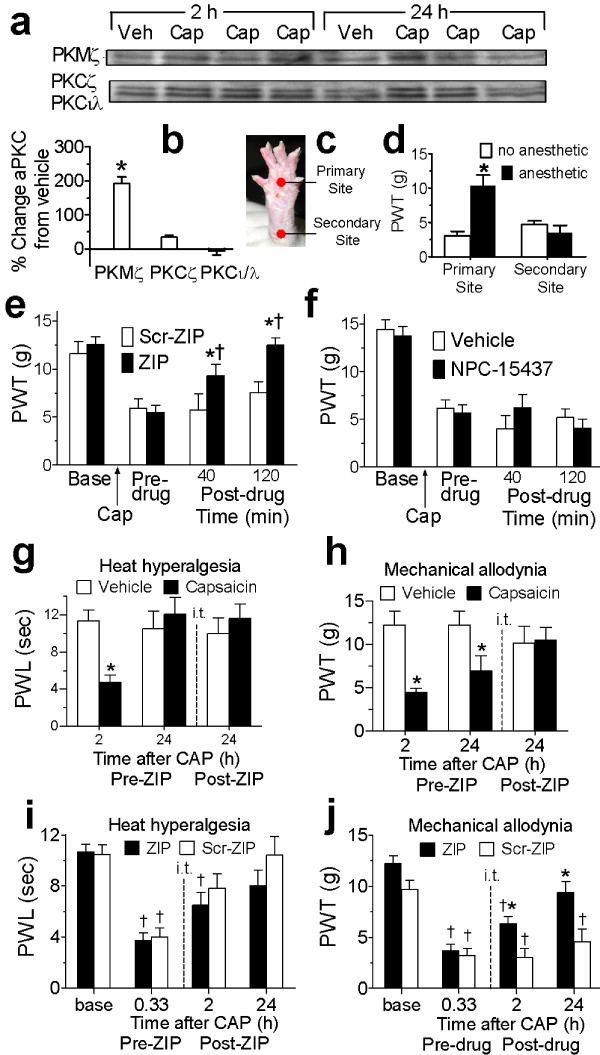
***SCDH atypical PKC expression, and effects of PKMζ/PKC inhibition on capsaicin-induced allodynia***. (**a**) Western blots and (**b**) histograms (% change) of atypical PKC showing i.pl. capsaicin increased PKMζ, but not PKCζ or PKCι/λ, in SCDH from 2-24 h (*P < 0.05, n = 7/12 vehicle/capsaicin). **c**) Primary/secondary allodynia test sites in capsaicin-injected rats. **d**) Paw-withdrawal thresholds (PWTs) 24 h post-capsaicin after anesthesia (n = 5) or no-anesthesia (n = 5) of the primary site. Primary, but not secondary, allodynia was attenuated by anesthesia (*P < 0.05, from no-anesthesia). (**e,f**) Pre- and post-capsaicin PWTs (g) with ZIP, scr-ZIP, NPC-15437 or vehicle (n = 15,4,6,6, respectively) given 24 h post-capsaicin. ZIP (*P < 0.05), but neither scr-ZIP nor NPC-15437, significantly reversed capsaicin-induced allodynia 40-120 min post-drug. (**g,h**) Effect of ZIP given 20 min before testing on paw-withdrawal latencies, PWLs (s) and PWTs (g) 24 h after capsaicin or vehicle (n = 6,4, respectively). **g**) Heat hyperalgesia present at 2 h post-capsaicin (*P < 0.05, from vehicle), abated by 24 h, and PWLs were unaffected by ZIP. **h**) Mechanical allodynia 24 h post-capsaicin (*P < 0.05 from vehicle) was reversed by ZIP. (**i,j**) Effect of ZIP or scr-ZIP (n = 11, 8, respectively) 100 min post-capsaicin on PWLs and PWTs 20 min, 2 and 24 h post-capsaicin. **i) **Heat hyperalgesia (20 min-2 h, †*P *< 0.05 from baseline), abated by 24 h, and was unaffected by ZIP at 2-24 h post-capsaicin. **j) **Mechanical allodynia was significantly attenuated at 2 h, and reversed at 24 h, post-capsaicin by ZIP (*P < 0.05 from scr-ZIP).

We first examined whether cutaneous injuries that induce persistent pain produced changes in expression of the 3 atypical PKC isozymes, PKMζ, PKCζ and PKCι/λ, in rat SCDH using an antibody raised against the C-terminal PKCζ that recognizes all three species [12]. Rats were given an intraplantar (i.pl.) hind paw injection of 2.0% formalin, 0.1% capsaicin or vehicle, and PKMζ, PKCζ and PKCι/λ were assayed by Western blot in SCDH homogenates at various time points after injury. Compared to levels in vehicle-treated rats, PKMζ, as well as PKCζ and PKCι/λ expression were substantially increased in SCDH 45 min after formalin (Figure 1a,b), but only PKMζ was increased 2-24 h after capsaicin (Figure 2a,b) injection. Thus, injuries causing persistent nociception produce sustained increases in spinal PKMζ, but transient increases (< 2 h) in PKCζ and PKCι/λ.

### Spinal PKMζ contributes to persistent pain after cutaneous injury

We next compared the effects of intrathecal (i.t.) pre- or post-treatment of a cell membrane-permeable PKMζ inhibitor myristoylated-ζ-pseudosubstrate inhibitory peptide (ZIP), or its scrambled control peptide (scr-ZIP), on persistent pain induced by i.pl. formalin. We have previously shown that persistent "late phase" formalin pain depends largely on spinal plasticity that occurs in response to pain during an acute early phase [27]. Here we compared the effects of ZIP with the PKC inhibitor NPC-15437, which inhibits all PKC isoforms except PKMζ, since it binds within the regulatory domain of PKC [28] (which is absent in PKMζ). Compared to scr-ZIP, *pretreatment *with 0.5-20 nmoles of i.t. ZIP significantly reduced persistent pain, but not acute formalin pain (Figure 1d). A dose of 10 nmoles was used subsequently since it had no significant effects on motor coordination in the rotarod test (Figure 1c), or on nociceptive thresholds to heat or mechanical stimulation in naïve rats (Figure 1f,g). *Post-treatment *with 10 nmoles i.t. ZIP, but not scr-ZIP, also significantly reversed persistent formalin pain when given 25 min post-formalin (Figure 1h). Further, despite analgesia in the formalin test induced by pretreatment with the full-length PKC inhibitor NPC-15437 (Figure 1e), post-treatment with NPC-15437 had no effect on persistent formalin pain (Figure 1i). Thus, unlike PKMζ, full-length PKCs are likely involved in inducing, but not sustaining, the nociceptive plasticity underlying persistent formalin pain. Moreover, together with our biochemical results, these data indicate that although both PKC (ζ and ι/λ) and PKMζ are increased by formalin, the sustained plasticity that underlies persistent late pain depends on PKMζ.

### Spinal PKMζ contributes to pain hypersensitivity after cutaneous injury

We next determined the effects of PKMζ inhibition on pain hypersensitivity induced by hind paw capsaicin injection. Capsaicin was used to distinguish the separate contributions of acute peripheral nociceptor sensitization and persistent central neuroplasticity to pain hypersensitivity. In both humans [29] and rats [30], skin injury with capsaicin produces spontaneous pain lasting ~15 min, and subsequently an early (2 h) localized primary heat hyperalgesia that depends on peripheral nociceptor sensitization, and a persistent (~24 h), wide-spreading secondary mechanical allodynia (an important feature of chronic pain) that for humans has been shown to depend on central neuroplasticity [29]. We show secondary mechanical allodynia 24 h after intradermal (i.d.) capsaicin injection in rats likely also depends on central neuroplasticity, as it is maintained after local anesthesia of the capsaicin injection site (Figure 2c,d). We hypothesized that if PKMζ is involved in sustaining spinal plasticity underlying persistent pain then i.t. ZIP should reverse the secondary mechanical allodynia even when given well after the injury. Importantly, i.t. ZIP, but not scr-ZIP, reversed secondary mechanical allodynia when given 24 h post-capsaicin (Figure 2e). In contrast, the full-length PKC inhibitor, NPC-15437, was unable to reverse secondary mechanical allodynia (Figure 2f) when given at the same time point. Thus, spinal PKMζ, but not full-length PKCs, is involved in sustaining spinal plasticity underlying persistent secondary pain hypersensitivity. Furthermore, the mechanical allodynia (at 2 and 24 h post-capsaicin), but not peripheral nociceptor sensitization-mediated, heat hyperalgesia (at 2 h post-capsaicin) was attenuated by i.t. ZIP, but not scr-ZIP, given early (100 min) or late (24 h) post-capsaicin (Figures 2g-j). Thus, ZIP reversed the persistent mechanical allodynia dependent on central sensitization, but not acute heat hyperalgesia dependent on peripheral nociceptor sensitization. Although ZIP treatment attenuated mechanical allodynia at 2 h (Figure 2j), it likely did not completely reverse it, because there was still an important contribution of primary sensitization at this point (note that testing was not performed separately at primary and secondary sites in this trial to due a larger capsaicin injection volume needed to induce heat hyperalgesia).

### Spinal PKMζ contributes to capsaicin-induced sensitization of WDR neurons

**Figure 3:**
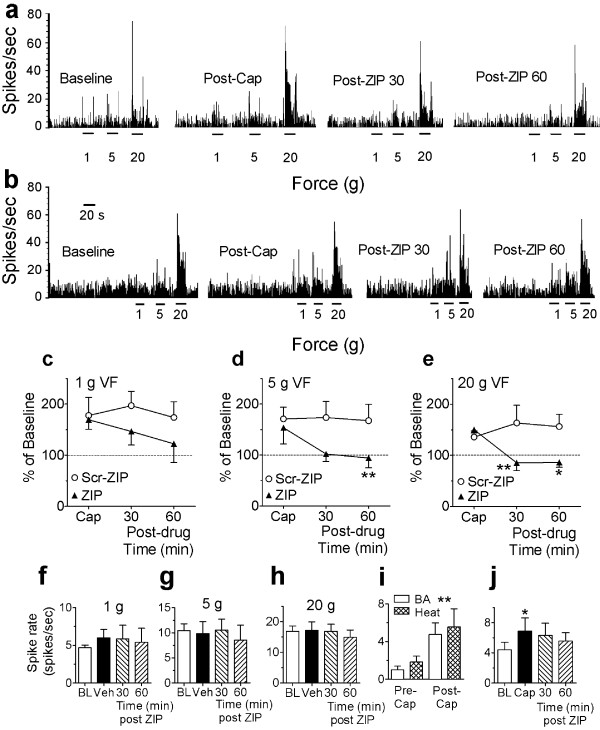
***Effects of PKMζ inhibition on capsaicin-induced sensitization of spinal WDR neurons***. (**a,b**) Spike rate (spikes/sec) of evoked responses of 2 WDR neurons to von Frey filaments pre- and post-capsaicin. ZIP (90 min post-capsaicin) reduced the capsaicin-induced sensitization of mechanically-evoked responses of the neuron in (**a**). The neuron in (**b**) was not sensitized by capsaicin, and ZIP did not reduce evoked responses to the mechanical stimuli. (**c-e**) Mechanically-evoked WDR neuron responses to 1 (**c**), 5 (**d**) or 20 (**e**) g stimuli pre- and post-capsaicin (% of baseline action potentials evoked/20 s). ZIP, but not scr-ZIP (n = 6, 5, respectively), significantly reversed capsaicin-enhanced evoked responses to 5-20 g stimuli at 30-60 min post-drug (*P < 0.05). (**f-h**) Mechanically-evoked WDR neuron responses to 1 (**f**), 5 (**g**) or 20 (**h**) g stimuli pre- (baseline, BL) and post-vehicle (before (Veh) and after ZIP (30-60 min) applied 90 min post-vehicle) (n = 5). Neither i.pl. vehicle, nor i.t. ZIP, significantly affected evoked responses of WDR neurons. **i) **Background activity and evoked responses to heat pre- and post-capsaicin. Although capsaicin significantly increased the background activity of WDR neurons (** P < 0.01 from Pre-capsaicin), it failed to increase their heat-evoked responses (*P >*0.05, n = 8). **j) **Background activity of WDR neurons pre- (baseline, BL) and post-capsaicin (before (Cap) and after ZIP (30-60 min) applied 90 min post-capsaicin, n = 6). Capsaicin significantly increased background activity (*P < 0.05), and ZIP failed to significantly reduce the capsaicin-induced increase in the background activity of WDR neurons (*P *> 0.05).

We also show that spinally-administered ZIP, but not scr-ZIP, when given 90 min after hind paw capsaicin, reversed the sensitization of spinal wide-dynamic-range (WDR) neurons to mechanical stimulation (Figure 3a,c-e). Importantly, ZIP had no effect on a WDR neuron that was not sensitized by capsaicin (Figure 3b), or when given after hind paw vehicle injection which does not sensitize WDR neurons (Figure 3f-h), indicating that ZIP reverses spinal sensitization, but does not simply suppress all stimulation-evoked activity. Although overall background activity of WDR neurons was increased by hind paw capsaicin injection, we did not see an increase in the evoked responses to noxious heat (Figure 3i). Furthermore, ZIP failed to reduce the significant capsaicin-induced increase in background activity of WDR neurons (Figure 3j).

### Spinal PKMζ is upregulated and contributes to pain hypersensitivity after spinal stimulation

**Figure 4:**
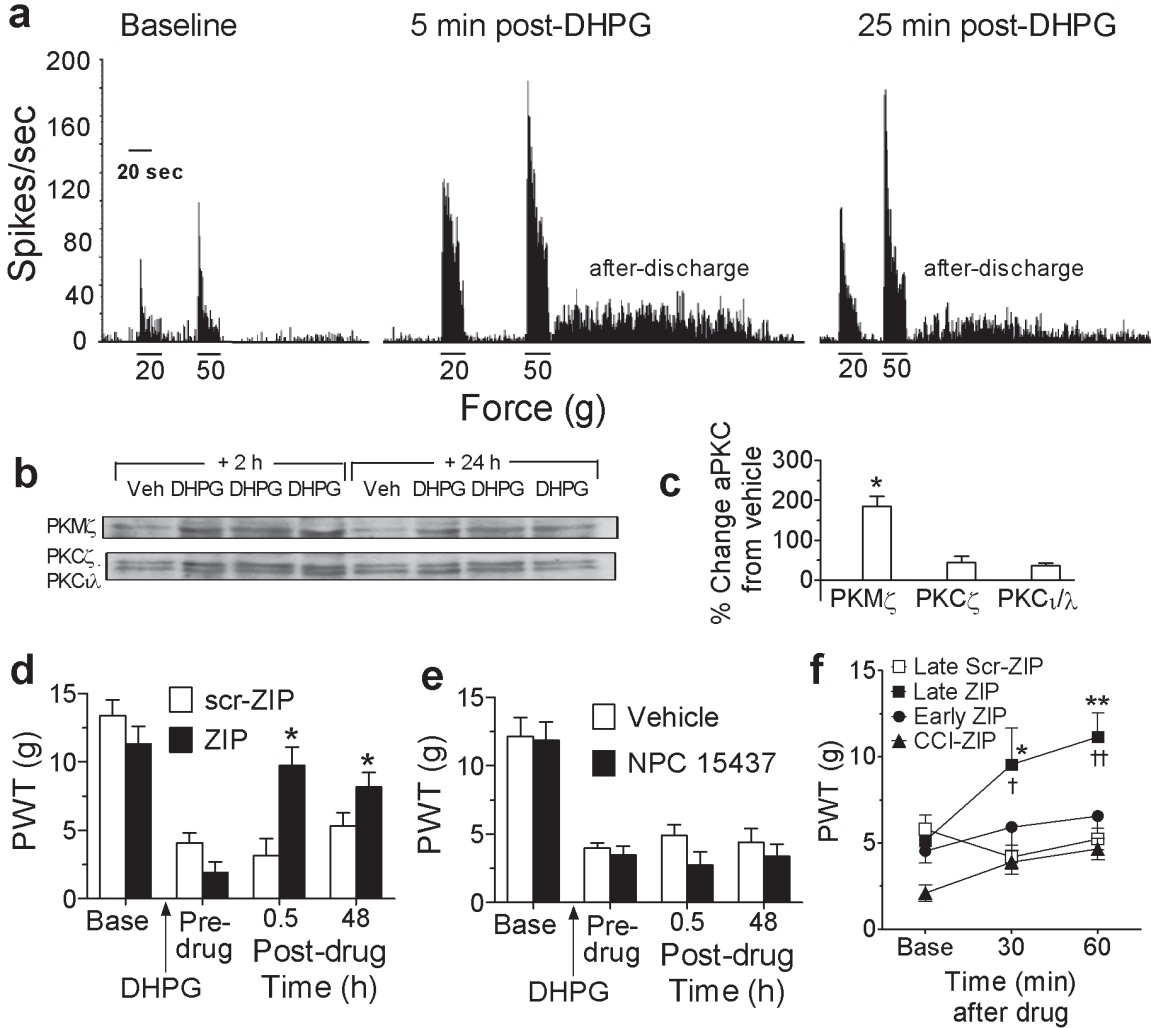
***SCDH atypical PKC expression, and effects of PKMζ/PKC inhibition on spinal DHPG-induced allodynia***. (**a**) Spike rate (spikes/sec) of evoked responses of a WDR neuron to 20 and 50 g von Frey filaments before and 5 or 25 min after DHPG. Note the increased evoked responses to both mechanical stimuli, and the prolonged after-discharges to the 50 g stimulus, both 5 and 25 min post-DHPG. Scale bar (20 sec). (**b**) Western blots and (**c**) histograms of % change in atypical PKC levels compared to vehicle illustrating that i.t. DHPG significantly increased PKMζ (n = 12), but not PKCζ (n = 12) or PKCι/λ (n = 8) in SCDH (vehicle, n = 11), 2 and 24 h post-DHPG combined (*P < 0.05). (**d,e**) Pre- and post-DHPG paw withdrawal thresholds (PWTs, g) for rats given i.t. ZIP or scr-ZIP (**d**), i.t. NPC-15437 or vehicle (**e**), 30 min prior to testing at 24 h post-DHPG, with additional testing 48 h post-drug. ZIP (*P < 0.05), but not scr-ZIP or NPC-15437, reversed DHPG-induced mechanical allodynia at both time-points (n = 5 for each drug). **f**) Pre-drug (base) and post-drug PWTs of CCI rats (n = 8), early CPIP rats (n = 9) and late CPIP rats treated with i.t. ZIP (n = 7), or late CPIP rats treated with i.t. scr-ZIP (n = 8). ZIP, but not scr-ZIP, reversed allodynia in late CPIP rats, while ZIP had no effects in CCI or early CPIP rats (* P < 0.01; ** P < 0.001 from scr-ZIP; † P < 0.05; †† P < 0.01 from pre-drug baseline).

We next examined whether i.t. ZIP affected persistent pain hypersensitivity induced by direct activation of spinal neurons with the metabotropic glutamate receptor agonist dihydroxyphenylglycine (DHPG). We have previously shown that i.t. DHPG produces acute pain (~60 min), followed by persistent mechanical allodynia [31]. Here we show i.t. DHPG sensitized a spinal WDR neuron to mechanical stimuli (Figure 4a), and increased PKMζ, but not PKCζ or PKCι/λ, in SCDH at 2-24 h (Figure 4b,c). Furthermore, mechanical allodynia induced 24-72 h after 10 nmoles of i.t. DHPG was reversed by i.t. ZIP, but not scr-ZIP, given 20 min before the 24 h test (Figure 4d). Once again these effects could not be replicated by inhibition of spinal full-length PKCs (Figure 4e), stressing the specific role of PKMζ in sustaining spinal plasticity; this time after direct spinal stimulation.

### Inhibition of spinal PKMζ reverses mechanical allodynia in CPIP, but not CCI rats

Finally, we assessed the effects of PKMζ inhibition in animal models of chronic pain, including chronic post-ischemia pain (CPIP) and chronic constriction injury (CCI) of the sciatic nerve. We tested the hypothesis that chronic models for which ongoing inputs from peripheral pathology are critical for allodynia would be less sensitive to the anti-allodynic effects of spinal PKMζ inhibition, than those in which the allodynia depends more on central changes. Thus, rats with CCI have allodynia dependent on persistent ongoing inputs from the constricted sciatic nerve [32,33], whereas CPIP rats initially have allodynia dependent on peripheral pathology (microvascular dysfunction and lactate accumulation in muscle), and later, once microvascular dysfunction resolves between 2-3 weeks, have continuing allodynia that is dependent on spinal changes [34]. We show that allodynia in late CPIP rats (3 weeks post-injury) is significantly alleviated by i.t. ZIP, but not scr-ZIP (Figure 4f). In contrast, allodynia in CCI rats, or in early CPIP rats (3-4 days post-injury), is not significantly affected by i.t. ZIP (Figure 4f).

## Discussion

Full-length PKCs in SCDH have previously been shown to contribute to the *induction *of pain hypersensitivity, or allodynia and hyperalgesia and neuronal sensitization that are driven largely by continued inputs from the peripheral site of injury [4,5,8]. The present results, however, indicate the autonomous PKC isoform, PKMζ, plays a specific essential role in *sustaining *spinal plasticity underlying persistent pain. PKMζ was persistently increased in SCDH by cutaneous injuries (i.pl. formalin and capsaicin), or by spinal stimulation with a glutamate agonist, each of which produces persistent pain or long-lasting pain hypersensitivity that is selectively reversed by inhibition of PKMζ, but not full-length PKCs. Additional shorter-term increases in full-length PKCζ and PKCι/λ 45 min after i.pl. formalin injection are consistent with the behavioral data suggesting a role of full-length PKCs in the induction of persistent pain. However, at later time points (2 and 24 h) after i.pl. capsaicin or i.t. DHPG, only PKMζ is elevated, indicating that SCDH PKMζ specifically is persistently elevated in association with the *maintenance *of persistent pain.

The specificity of PKMζ effects in sustaining spinal plasticity underlying persistent pain is highlighted by the ability of i.t. treatment with a selective PKMζ inhibitor to reverse established persistent pain or pain hypersensitivity, while treatment with a full-length PKC inhibitor fails to do so, despite its ability to prevent the induction of persistent pain. This transition from the transient role of full-length PKCs to the persistent function of autonomously active PKMζ in pain is reminiscent of similar transitions in LTP and memory consolidation [13-17]. Furthermore, the finding that spinal PKMζ inhibition not only reversed hind paw capsaicin-induced allodynia, but also hind paw capsaicin-induced sensitization of SCDH WDR neurons (evoked by the same mechanical stimuli) provides further support for the role of PKMζ in the maintenance of spinal nociceptive plasticity. Earlier studies demonstrating that pretreatment [25], but not post-treatment [26] with NPC-15437 reduced capsaicin-induced sensitization of spinal neurons to mechanical stimulation, is also consistent with our behavioral data showing that post-treatment with NPC-15437 does not reverse persistent pain or allodynia after cutaneous injury or spinal stimulation. Importantly, this earlier study used the same PKC inhibitor we employed, which does not inhibit PKMζ.

Our results are consistent with and extend upon three recent papers that have examined the role of PKMζ in nociception [21,22,35]. The first paper showed that PKMζ was increased within the anterior cingulate cortex (ACC) of neuropathic mice, and that PKMζ inhibition in this brain area, but not SCDH, produced anti-allodynic effects in neuropathic mice [21]. A role of PKMζ in nerve-injury induced neuronal plasticity in ACC was further supported since ZIP reduced α-amino-3-hydroxy-5-methyl-4-isoxazole-propionate (AMPA) receptor-mediated excitatory post-synaptic potentials (EPSPs) in ACC neurons of nerve-injured, but not sham mice. Additionaly, PKMζ inhibition reduced spinal neuron AMPA receptor-mediated EPSPs in nerve-injured, as well as sham mice [21]. Importantly, these electrophysiological experiments were performed in brain and spinal cord slices, where there are no longer ongoing inputs from the injured nerves. However, the lack of effect of spinal PKMζ inhibition on allodynia does not rule out a role for PKMζ in spinal plasticity, if the inhibitor is unable to overcome the effects of ongoing inputs from injured nerves. Thus, our results, showing a lack of anti-allodynic effect of spinal ZIP in CCI rats, support the findings of Li et al. [21] in neuropathic mice. However, we found that i.t. ZIP relieved allodynia in late CPIP rats, in which allodynia depends on central changes (as peripheral pathology has resolved), but not for early CPIP rats, in which allodynia depends on ongoing peripheral inputs [34]. This suggests that while PKMζ may contribute to spinal plasticity in these models, the ability of spinal PKMζ inhibition to relieve allodynia is overshadowed in CCI rats or early CPIP rats when there is significant ongoing peripheral input.

The second paper showed that inhibition of PKMζ in spinal cord prevented the enhancement of allodynia (in response to cutaneous injury) or persistent pain (in response to i.t. DHPG) that occurred following an earlier resolved cutaneous injury (either i.pl. IL-6 injection or plantar incision) [22]. In this case, PKMζ inhibition was shown to prevent the development of enhanced responses to nociceptive stimulation in situations where an earlier injury results in nociceptive priming. Our results support this earlier study's conclusions, extending them to include ZIP's ability to *reverse *an established persistent pain or pain hypersensitivity. Importantly, Asiedu et al. [22] did not reverse allodynia, but rather prevented allodynia induced by a second injury. Together the two studies strongly implicate the role of PKMζ in maintaining spinal nociceptive plasticity for both nociceptive priming and persistent pain.

Our data is also consistent with a recent third report published in this journal showing that PKMζ activity (specifically localized in spinal projection neurons) is increased after formalin injury, and that a PKMζ inhibitor reduced nociceptive behaviors, WDR neuronal activity and Fos protein levels in SCDH in response to hind paw formalin injection [35]. However, these investigators only examined the effects of *pretreatment *with ZIP on formalin-induced nociceptive and dorsal horn neuronal responses. While data here and in this previous study implicate a role of PKMζ in the *induction *of nociceptive plasticity; the current study, showing a post-treatment reversal of formalin-induced persistent nociception, as well as allodynia and WDR sensitization induced by hind paw capsaicin, and allodynia induced after i.t. DHPG, or in late CPIP rats, also implicates PKMζ in the *maintenance *of spinal nociceptive plasticity in persistent pain.

Marchand et al. [35] also showed that spinal PKMζ inhibition was unable to reduce mechanical hypersensitivity in neuropathic animals, although they did show that ZIP post-treatment attenuated mechanical hypersensitivity in rats with complete Freund's adjuvant (CFA)-induced hind paw inflammation. However, the effect on mechanical hypersensitivity was only partial and short-lived (30 min), with slightly better effects on thermal hypersensitivity. These investigators attributed the differential effects of ZIP on neuropathic and inflammatory pain to disparate molecular mechanisms of nociceptive plasticity underlying these two injuries. However, based on the differential effects of ZIP in CCI and early CPIP rats vs. late CPIP rats observed in our study, the disparate results could also be explained by differences in the contribution of ongoing inputs from injured peripheral tissue, which are particularly enhanced in neuropathic animals [32,33], and we expect are reduced in late CPIP rats when the injured tissue has healed [34]. While it is true that there may be ongoing peripheral inputs in CFA rats, it could also be argued that the high levels of spontaneous C- and Aδ- and A-β fiber activity in damaged peripheral nerves in CCI rats (generated both at the site of injury and at the dorsal root ganglion [32,33]) may represent greater peripheral drive. Furthermore, peripheral drive from inflamed CFA hind paws may explain the partial and short-lived effects of i.t. ZIP on mechanical and thermal hypersensitivity in these animals.

## Conclusions

The present results implicate spinal PKMζ as a key mediator sustaining plasticity in SCDH underlying persistent pain. Thus, persistent pain, pain hypersensitivity and the sensitization of SCDH neurons after cutaneous injury, or chronic allodynia after ischemia injury, all depend on PKMζ for their maintenance. The specific role of PKMζ in sustaining spinal nociceptive plasticity contrasts with the role of full-length PKCs, which appears to play a role only in its induction. The results suggest that spinal PKMζ may be an important new target for the development of pharmacological therapies for chronic pain.

## Methods

### Subjects

Subjects were male Long-Evans hooded rats obtained from Charles River (Senneville, QC). The rats were housed 3 to a cage and were maintained on a 12 h light/dark cycle with food and water available *ad libitum*. All procedures were carried out during the light cycle, and with approval of the McGill University Animal Care Committee.

### Injuries or stimuli inducing allodynia and hyperalgesia

Cutaneous injuries were induced by intraplantar (i.pl.) injections of either formalin (Sigma, 2.0%, 50 μl, in 0.9% saline) or capsaicin (Sigma, 0.1%, 20 μl for trials with heat hyperalgesia or 0.5%, 10 μl intradermally (i.d.) for trials with secondary mechanical allodynia, in 7.5% Tween 80/saline). Direct spinal stimulation was produced by intrathecal (i.t.) injection of 10 nmoles of dihydroxyphenylglycine (DHPG, Tocris, in 20 μl) [31]. Within 45 min post-formalin, or for periods between 2-72 h post-capsaicin or post-DHPG, rats were subjected to behavioral nociceptive testing or SCDH electrophysiological recording, or had their spinal cords removed by pressure ejection following sodium pentobarbital anesthesia and decapitation.

Neuropathic injury was induced by chronic constriction injury (CCI) of the sciatic nerve, as described by Bennett and Xie [36]. Briefly, while rats were under sodium pentobarbital anesthesia, the sciatic nerve was exposed at mid-thigh level and 4 ligatures (4.0 chromic gut; Ethicon) were loosely tied around the nerve 2 mm apart, to produce a loose constriction of the nerve. Chronic post-ischemic pain was induced by 3 h of hind paw ischemia followed by reperfusion, as we have done previously [34,37]. Briefly, rats were anesthetized over a 3 h period with a bolus (55 mg/kg, i.p.) and chronic i.p. infusion of sodium pentobarbital for 2 h (27.5 mg/kg/h for two hours). After induction of anesthesia, a Nitrile 70 Durometer O-ring (O-rings West, Seattle, WA) with 7/32 in internal diameter was placed around the rat's left ankle joint for 3 h, and then removed to allow reperfusion.

### Western Blotting

Proteins (15 μg total protein per lane) from SCDH samples homogenized in RIPA buffer were separated by gel electrophoresis (SDS-PAGE) on a 9% polyacrylamide gel, and electrotransferred to PVDF membrane. The membranes were probed with a primary antibody raised to the C-terminal of atypical PKCs (Santa Cruz SC-216; 1:400) [12], which recognizes PKCζ (72-76 kDa), PKMζ (50-55 kDa) and PKCλ/ι (68-70 kDa). The antibody was incubated with peroxidase-coupled secondary antibody (NA 934, GE Life Sciences; 1:30,000), followed by incubation with chemiluminescent substrate (RPN 2132, GE Life Sciences). Developed blots were scanned with a Canon N650U Scanner and analyzed with ImageJ 1.44p software from NIH.

### Intrathecal Drug Administration

Drugs were administered to the intrathecal (i.t.) space either by lumbar puncture or through chronic indwelling catheters (formalin test only), while rats were anesthetized with isoflourane (4% induction, 2% maintenance). For lumbar punctures, a 27 gauge needle was inserted between the L5/L6 vertebrae into the *cauda equina *and drugs were administered in a 20 μl volume. I.t. placement was verified by a flick of the tail on needle entry and injection. For indwelling catheters, a lumbar puncture (L5/L6) was performed using a 23 gauge needle, and PE-32 polyurethane tubing was pushed 3 cm through the needle to the lumbar enlargement, before the needle was removed [38]. Injections were given to conscious rats and i.t. placement was verified by temporary hind paw paralysis following lidocaine injection (20 μl, 2%) performed immediately after behavioral testing, and the data from rats unaffected by the lidocaine were not used. ZIP (Myr-SIYRRGARRWRKL-OH, Tocris), scr-ZIP (Myr-RLYRKRIWRSAGR-OH, Tocris), NPC-15437 (Sigma) and DHPG were dissolved in 100 mM Tris-saline (pH 7.2).

### Behavioral Testing

Naïve rats and rats given i.pl. injections of 20 μl of 0.1% capsaicin were tested for potential analgesic/anti-hyperalgesic/anti-allodynic effects, or for the presence of either heat and mechanical hyperalgesia/allodynia (at the injection site for capsaicin-injected rats) by measuring heat or mechanical nociceptive flexion responses. A nociceptive flexion response to heat was quantified using the plantar test [39]. After the rat was habituated to the Plexiglas^® ^chamber in a Hargreaves apparatus, the plantar surface of the rat's affected hind paw was exposed to a noxious radiant heat source from beneath the floor, and the time was measured until the rat moved its hind paw. For each test, three measurements were obtained from each animal, spaced approximately 10 min apart. The intensity was set at a level that normally elicited a response within 10-12 sec. A mechanical flexion response was quantified using von Frey filaments. After the rat was habituated to a testing box with a wire-mesh floor, a series of von Frey filaments (0.41-15.1 g) were applied to the ventral surface of the rat's hind paw. Filaments were presented using an up-down procedure, and a 50% response threshold was calculated for each rat, as previously described [40]. Rats given i.t. injections of DHPG were tested for mechanical allodynia in the hind paw using von Frey filaments as indicated above.

To assess secondary mechanical allodynia, rats were tested with von Frey filaments as above, except that mechanical allodynia was measured at a secondary site at the heel, approximately 10 mm from the intradermal (i.d.) injection of 10 μl of 0.5% capsaicin immediately proximal to the anterior two foot pads, similar to the method used by Kinnman and Levine [41] (Figure 2c). Secondary mechanical allodynia was assessed in i.t. drug trials, together with the assessment of primary allodynia at the capsaicin (primary) injection site, with or without local anesthesia (10 μl, 2% lidocaine, i.d.) of the primary site.

Persistent painful responses to a 50 μl i.pl. hind paw injection of 2.0% formalin were assessed using weighted means behavioral pain scores as previously described [27]. Briefly, a pain score was determined for 1 min of observation during each 3 min block by measuring the amount of time spent in each of 4 behavioral categories: 0, the injected paw is not favored; 1, the injected paw has little or no weight on it; 2, the injected paw is elevated and is not in contact with any surface; 3, the injected paw is licked, bitten or shaken. The time spent in each category was then multiplied by its category weight, summated, and divided by the total time the rat was observed within each time block. The early phase was defined as responses during the first two 3 min time blocks, and the late phase as responses between 9 and 45 min post-formalin injection. Drugs were given i.t. by chronic indwelling catheter as pretreatments 20 min prior to the formalin injection, or as post-treatments 25 min post-formalin injection, i.e., once the late phase had been established.

For rotarod testing, rats were given 6 daily training trials for 5 days and 6 post-drug test trials on day 6 on a rotarod (IITC model 730), with trials spaced 12 min apart. Each trial lasted a maximum of 120 s, during which time the rod rotation increased from 0 to 30 rpm. The latency to fall off the rod was recorded, and latencies for the 6 trials on each day were averaged. On the sixth day, each rat was given i.t. ZIP (10 nmol/20 μl) 20 min before the start of the trials.

### Electrophysiology

For electrophysiology trials, capsaicin (30 μl, 0.1%, i.pl.) or vehicle was injected in the receptive field of identified WDR neurons in the SCDH of 27 anesthetized rats, as previously described [42]. Spontaneous spike frequency and spikes evoked in WDR neurons in response to mechanical stimulation with von Frey filaments (1-50 g) were recorded pre-capsaicin (or vehicle), 30-60 min post-capsaicin/vehicle and 30-60 min post-drug. Evoked responses of WDR neurons to noxious heat (a feedback-controlled halogen heat lamp focused on the hind paw which heated the skin to 60°C) were also examined pre- and post-capsaicin. Capsaicin/vehicle was injected inside the receptive field of each neuron, but distal to the sites of mechanical or thermal stimulation. ZIP, or scr-ZIP (50 nmoles in 100 μl), was administered by spinal application 90 min after capsaicin/vehicle. Stimulus-evoked responses were defined as the total number of action potentials (spikes) evoked during 20 s of stimulation. Similar electrophysiological methods for mechanical evoked responses were used in an additional rat that received spinal application of DHPG (50 nmoles in 100 μl) instead of i.pl. capsaicin.

### Statistical analyses

All protein assays and behavioral data were analyzed using either one-way independent groups or two-way repeated measures ANOVA followed by orthogonal contrasts or multiple comparisons with Fisher's test. Orthogonal contrasts were used in the formalin test when the number of potential comparisons was far greater than the comparisons of interest. Electrophysiological data converted to percent change from baseline was analyzed using Friedman's ANOVA, and raw spike rate data was analyzed using ANOVA, each followed by multiple comparisons with Dunn's test. Percent change scores were used because we have previously found that they remain stable across large variations in baseline neuronal activity [42]. In all tests, the criterion for statistical significance was *P *< 0.05.

## Competing interests

The authors declare that they have no competing interests.

## Authors' contributions

AL performed behavioral experiments and protein assays, analyzed the results and prepared figures. MHP completed all electrophysiological experiments. YH, AH and VC assisted with behavioral testing. NK performed surgeries to implant i.t. catheters, and FC participated in the development of electrophysiology experiments. TCS assisted in experimental interpretation and editing the manuscript. TJC conceived all experiments and wrote the manuscript. All authors have read and approved the final manuscript.
